# Clinical deployment environments: Five pillars of translational machine learning for health

**DOI:** 10.3389/fdgth.2022.939292

**Published:** 2022-08-19

**Authors:** Steve Harris, Tim Bonnici, Thomas Keen, Watjana Lilaonitkul, Mark J. White, Nel Swanepoel

**Affiliations:** ^1^ Institute of Health Informatics, University College London, London, United Kingdom; ^2^ Department of Critical Care, University College London Hospital, London, United Kingdom; ^3^ Centre for Advanced Research Computing, University College London, London, United Kingdom; ^4^ Digital Healthcare, University College London Hospital, London, United Kingdom

**Keywords:** translational medicine, machine learning, health informatics, ML-Ops, safety, artificial intelligence

## Abstract

Machine Learning for Health (ML4H) has demonstrated efficacy in computer imaging and other self-contained digital workflows, but has failed to substantially impact routine clinical care. This is no longer because of poor adoption of Electronic Health Records Systems (EHRS), but because ML4H needs an infrastructure for development, deployment and evaluation within the healthcare institution. In this paper, we propose a design pattern called a Clinical Deployment Environment (CDE). We sketch the five pillars of the CDE: (1) real world development supported by live data where ML4H teams can iteratively build and test at the bedside (2) an ML-Ops platform that brings the rigour and standards of continuous deployment to ML4H (3) design and supervision by those with expertise in AI safety (4) the methods of implementation science that enable the algorithmic insights to influence the behaviour of clinicians and patients and (5) continuous evaluation that uses randomisation to avoid bias but in an agile manner. The CDE is intended to answer the same requirements that bio-medicine articulated in establishing the translational medicine domain. It envisions a transition from “real-world” data to “real-world” development.

## Introduction

Bold claims and huge investments suggest Machine Learning (ML) will transform healthcare (1). High impact publications showcase precision models that predict sepsis, shock, and acute kidney injury (2–4). Outside healthcare, tech titans such as AirBnB, Facebook, and Uber create value from ML despite owning “no property, no content and no cars” (5). Inspired by this, and very much aware of the flaws and unwarranted variation in human decision making (6), government and industry are now laying heavy bets on ML for Health (ML4H) (7, 8).

Widespread adoption of electronic health records (EHR) might be thought a sufficient prerequisite for this ambition. Yet while EHR adoption is growing at pace (9), those ML4H models that have reached the market rarely use the EHR. They are instead embedded in isolated digital workflows (typically radiology) or medical devices (10). Here the context of deployment is static and self-contained (imaging), or fully specified (devices), and translation has proved easier to navigate.

In contrast, the EHR is in constant flux. Both the data and the data model are updating. New wards open, staffing patterns are adjusted and from time to time major incidents (even global pandemics) disrupt everything. There are multiple interacting users, and eventually there will be multiple interacting algorithms, and organizations will face the ML equivalent of poly-pharmacy (11). Algorithms will require stewards (12). Whilst the aforementioned high impact prediction models are developed on real-world data, this is not the same as real-world development. Data are either anonymized and analyzed offline, or moved out of the healthcare environment into an isolated Data Safe Haven (DSH) [also known as Trusted Research Environment (TRE)] (13). This separation is the first fracture leading to the oft-cited AI chasm (14) leaving the algorithms stranded on the laboratory bench.

A future that sees ML4H generate value from the EHR requires an alternative design pattern. TREs excel at meeting the needs of population health scientists but they do not have the full complement of features required to take an ML4H algorithm from bench-to-bedside. Using drug development as an an analogy, a TRE is custom made for drug discovery not translational medicine (15).

In this paper, we describe the functional requirements for a Clinical Deployment Environment (CDE) for translational ML4H. These requirements map closely to the classical components of translational medicine, but differ in that algorithms will require ongoing stewardship even after a successful deployment. The CDE is an infrastructure that manages algorithms with the same regard that is given to medicines (pharmacy) and machines (medical physics). Moreover, the value of ML4H will not just be from externally developed blockbuster models, but will also derive from specific and local solutions. Our vision of a CDE therefore enables both *development* and *deployment*.

Our CDE is supported by five pillars:

1. Real World Development
2. ML-Ops for Health
3. Responsible AI in practice
4. Implementation science
5. Continuous evaluation

We describe these pillars below alongside figures and vignettes reporting early local experience in our journey building this infrastructure.

## 1. Real world development

Real-world data (RW-Data) means the use of observational data at scale augmented by linking across multiple data sources to generate insights simply not available from isolated controlled clinical trials (16). The FDA uses data from tens of millions of patients in its Sentinel programme to monitor drug safety, and the OpenSafely programme in the UK generated impactful insights into COVID-19 within the first few months of the global pandemic (17).

Given the sensitive nature of health data, these initiatives depend on expanding investment into TREs (18). TREs are an example of “data-to-modeler” (DTM) designs where data flows from source (primary, secondary, social care and elsewhere) to a separate, secure landing zone. Here research teams write the code to link, clean and analyze the data. Derived insights eventually return to the bedside through clinical guidelines and policy. To date, DTM is also the dominant design pattern in ML4H but this approach is fundamentally flawed.

It is flawed because it imposes a separation between the modeller and the end-user. ML4H is not concerned with better guidelines or policy but with better operational and clinical decision making. This requires the practitioner to work alongside the end-user because excellent offline model performance provides no guarantee of bedside efficacy. Algorithms with inferior technical performance may even provide greater bedside utility (19, 20). An inverted “modeler-to-data” (MTD) paradigm was initially proposed to reduce privacy concerns (data are no longer copied and shared but analyzed *in situ* (21)), but we see important additional value in that it forces “real-world development” (RW-Dev) and enables the end-user to work with the modeler in rapid-cycle build-test-learn loops. This first pillar of the CDE is the equivalent of an *internal* TRE *within* the healthcare institution (21).

RW-Dev has four functional sub-requirements that distinguish it from a TRE. (1) Firstly, data updates must match the cadence of clinical decision making. For most inpatient and acute care pathways, decisions are in real-time (minutes or hours) at the bedside or in the clinic. (2) Secondly, development using live data must be sandboxed and so the clinical system responsible for care delivery is protected (3) thirdly, privacy must be managed such that teams are able to develop end-user applications that inevitably display patient identifiable information (PII) alongside the model outputs: an anonymous prediction is of little use to a clinician. (4) Fourthly, attention must be paid to developer ergonomics. Where development and deployment steps are separated physically (the TRE paradigm) or functionally (*via* different languages and technologies), ownership is often split between two different teams. One team prepares the raw data and develops the model, and another prepares the live data and deploys the model. We argue instead that the same team should be able develop *and* deploy. This should accelerate iteration, reduce cost and increase quality (22).

**Figure 1:**
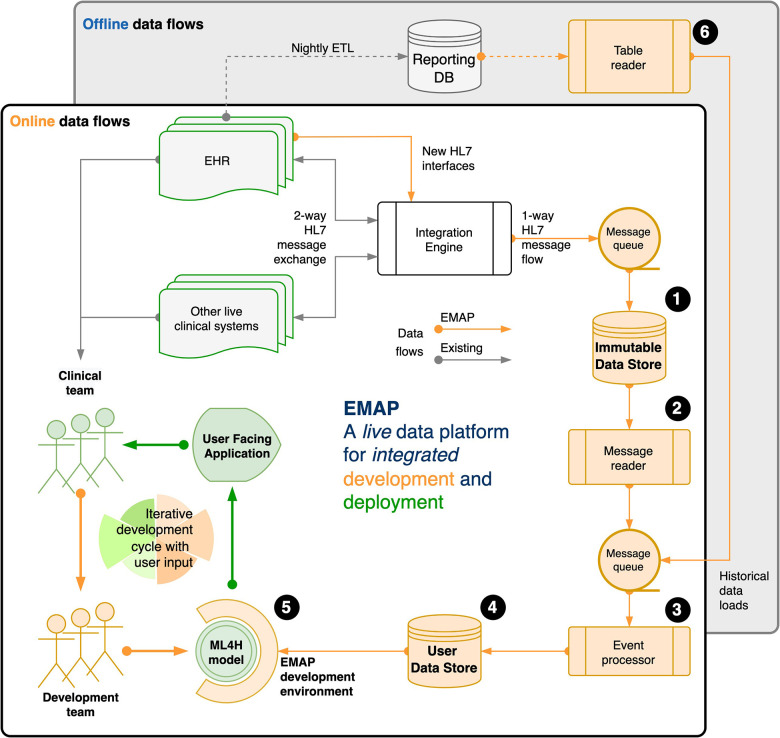
Our real-world development is performed on the Experimental Medicine Application Platform (EMAP). EMAP is a clinical laboratory within which ML4H researchers can iteratively build, test and gather feedback from the bedside. It unifies the data and the tools for off-line and online development of ML4H models (see figure and the **(numbers)** in the following sentences that refer to objects in the figure). In brief, EMAP builds a patient orientated SQL database from Health Level 7 version 2 (HL7v2) messages that are being exchanged between hospital systems. HL7v2 messages are ubiquitous in health care, and the de facto standard for internal communication. Rather than multiple pairwise connections between different hospital electronic systems, an integration engine acts as a single hub that routes HL7 messages, and where necessary translates to ensure compatibility. EMAP copies each message passing through the integration engine to a PostgreSQL database, the Immutable Data Store (IDS) **(1)**. A message reader **(2)** processes each live message to an interchange format so that downstream processing is insulated from local HL7 implementation. Separately, the table reader **(6)** processes historical data (e.g. from the reporting database) to the same interchange format. Live messages take priority over historical messages in a queue that feeds the event processor **(3)**. This links each message to a patient and a hospital visit, makes appropriate updates for out of order messages, and merges when separate identifiers are recognised to represent the same patient. A full audit trail is maintained. Each event updates a second live PostgreSQL database, the User Data Store (UDS) **(4)**. The hospital hosts Jupyter and RStudio servers, and a Linux development environment is provided that allows docker deployment, installation of analysis libraries and frameworks, exposes SSH and HTTPS services, and allows user verification against the hospital active directory. **(5)** A typical workflow might include investigation and experimentation in a Jupyter Notebook with data from the UDS, then using a small network of docker containers to run the development script, log outputs to a testing database, and report to users *via* email or a locally hosted web application or dashboard. A fuller explanation is available in the **Supplementary Material** (Section 2: EMAP data flows).

We illustrate this idea with a description of our local real-world development platform in Figure 1, and provide an extended description in the **Supplementary Material**.

## 2. ML-OPS (for health)

Hitherto in ML4H, the data and the algorithm have been the “celebrity couple”. State-of-the-art models trained on RW-Data deliver high profile publications (3, 4). But only a tiny handful (fewer than 10 studies in a recent high quality systematic review of nearly 2000 ML4H publications (23)), were prospectively implemented. The standard offline “data-to-modeler” (DTM) paradigm described above incurs a significant but “hidden technical debt” that includes configuration, data collection and verification, feature extraction, analysis and process tools, compute and storage resource management, serving infrastructure, and monitoring (24). In fact, the code for the underlying ML model is estimated to be at most 5% of the total code with the other 95% as additional code to make the system work. “Glue-code”, “pipeline jungles”, and “dead experimental codepaths” are some of the anti-patterns that make the transition into production costly and hazardous.^1^

Agencies such as the FDA, EMA, and MHRA are working toward safety standards for AI and machine learning, but the majority of these efforts derive from medical devices regulation. Treating Software as a Medical Device (SaMD) is appropriate where the algorithms operate within a constant and predictable environment (e.g. code embedded within a cardiac pacemaker). But, as already argued, ML4H models working with the EHR are likely to find themselves operating in a significantly more complex landscape. This inconstant environment where algorithms themselves may only have temporary utility has parallels to the commercial environment exploited so successfully by the tech giants.

**Figure 2:**
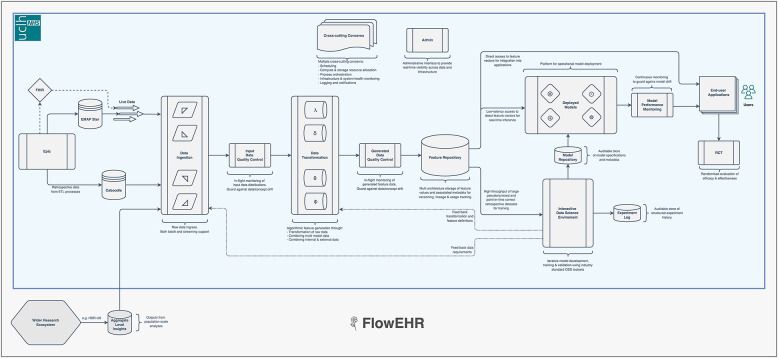
Our ML-Ops platform is called FlowEHR. Moving from left to right across the figure, the system monitors raw input data including checks for distribution shift, builds features with testable and quality controlled code, makes those features available to for both training and predictions to avoid train/serve skew, and maintains an auditable and monitored model repository.

These companies have cultivated an approach to model deployment called “ML-Ops”. This combines the practices of “DevOps” (a portmanteau of Software Development plus IT operations) (22) that focuses on the quality and speed with which software moves from concept to production, with robust data engineering and machine learning. A typical ML-Ops system monitors raw input data, checks for distribution drift, provides a feature store to avoid train/serve skew and facilitate collaboration between teams, and maintains an auditable and monitored model repository (26). We present a prototype implementation interacting with the EHRS in Figure 2 (called FlowEHR).

This constant adjustment of algorithms based on their continuously measured quality and performance needs a workforce as well as a technology stack. Just as the safe delivery of medicines to the bedside is the central activity of a hospital pharmacy team, the safe delivery of algorithms will require the development of similarly skilled and specialized practitioners, and we should expect to see clinical ML-Ops departments in the hospital of the future. Others have made similar proposals and labeled this as “algorithmic stewardship” or “AI-QI” (12, 27). Similarly, the FDA is now proposing “automatic Algorithmic Change Protocols” (aACP) and proposals have been advanced to guard against gradual deterioration in prediction quality (“biocreep”) (28, 29).

## 3. Responsible AI in practice

Pillars 1 and 2 should engender well designed and well engineered algorithms, but they do not protect against the unintentional harm that AI may induce. Algorithms can only learn from a digital representation of the world that representation in turn cannot encode moral or ethical standards. Unfair outcomes, discrimination against sub-populations and bias are all reported shortcomings (30). In a dynamic setting, risk can also arise in the form of degraded predictive performance over time. Models that modify clinician's behavior alter patient profiles by design, but predictive success today inevitably erodes future performance by rendering obsolete the historical patterns that drove the performance of the original model (31). Responsible AI in practice requires a systems approach that preempts and safe-guards against these potential risks to patients. We highlight three promising responses to components of this challenge that need to become part of the risk management approach for ML4H.

### 3.1. Model explainability

We argue that model explainability (Explainable Artificial Intelligence [XAI]) methods need to be prioritized to help systematize and coordinate the processes of model troubleshooting by developers, risk-management by service providers, and system-checks by auditors (32–35). Most AI models that operate as “black-box models” are unsuitable for mission-critical domains, such as healthcare, because they pose risk scenarios where problems that occur can remain masked and therefore undetectable and unfixable. We acknowledge recent critiques (36, 37) of explainability methods that argue the methods cannot yet be relied on to provide a determinate answer as to whether an AI-recommendation is correct. However, these methods do highlight decision-relevant parts of AI representations, and offer promise in measuring and benchmarking interpretability (38, 39). They are particularly promising for risk management as they can be used to structure a systematic interrogation of the trade-off between interpretability, model accuracy and the risk of model misbehavior.

### 3.2. Model fail-safes

Prediction models that map patient data to medically meaningful classes are forced to predict without the option to flag users when the model is unsure of an answer. To address this problem, there is good evidence that methods such as Bayesian deep learning and various uncertainty estimates (40) can provide promising ways to detect and refer data samples with high probability of misprediction for human expert review (41–43). These fail safes, or selective prediction approaches should be designed into support systems to preempt and mitigate model misbehavior (29, 44–47). Of note, the European Commission High-Level Expert Group on AI presented guidelines for trustworthy AI in April 2019 with such recommendations: for systems that continue to maintain human-agency *via* a human-in-the-loop oversight. This may even permit less interpretable models to operate when implemented in conjunction with an effective fail-safe system.

### 3.3. Dynamic model calibration

As discussed, models that influence the evolution of its own future input data are at risk of performance deterioration over time due to input data shifts (48). In such cases, continual learning *via* calibration drift detection and model recalibration (27, 49) provides a promising solution but remains a challenging paradigm in AI. Recalibration with non-stationary incremental data can lead to catastrophic forgetting when the new data negatively interferes with what the model has already learned (50), or a convergence where the model just predicts its own effect and thus should not be updated (31). On the other hand, models can propose poor decisions because of the inherent biases found within the original dataset. In this case, dynamic model recalibration is unlikely to be sufficient and larger model revisions may be required. Here Pillar 1 (RW-dev) with suitable audit and monitoring *via* Pillar 2 (ML-Ops) will be required to overcome what would otherwise be a learning process encumbered by regulatory barriers (51).

## 4. Implementation science

A well designed, safe, and responsible AI algorithm may still be ineffective if it does not reach a modifiable target on the clinical pathway (19). Unlike medications, algorithms can only effect health by influencing the behavior of clinicians and patients. This translational obstacle parallels the second arm of translational medicine (T2): implementation science (15). Behavior change, in most instances, will be *via* a modification of the choice architecture (passive) (52, 53) or *via* interruptive alerts (active) embedded in the EHR (53). Effective implementation requires a multi-disciplinary approach including human-computer interaction, behavioral science, and qualitative analysis (54).

We strongly argue that this task will be more difficult if done offline and in isolation. Pillar 1 crucially permits not just tuning of the technical performance of the algorithm but rapid build-test-learn cycles that directly involve the target user and the clinical pathway in question. This approach will reduce costs and improve impact, sometimes leading to trade-offs which might appear surprising to those developing away from the bedside (11, 20). This efficiency will again depend on the problem space: where the algorithmic target depends on information arising from the EHR rather than an isolated device or image, and where the pathway involves multiple end-users, then successful implementation will be near impossible if done sequentially (development then deployment) rather than iteratively (54, 55). Academic health science centres must become design “laboratories” where rapid prototyping at the bedside crafts the deployment pathway for *effectiveness* (T2) rather than just efficacy (T1) (15).

Investigations to define how system can influence behavior will need specialist support and tooling. This might require tools embedded within the user interface to evaluate and monitor user interaction, and capture user feedback (56), or directed implementation studies (57).

Despite the oft cited risks of alert fatigue with Clinical Decision Support Systems (CDSS) (58), there is good evidence that well designed alerts can be impactful (53, 59, 60). Overt behavioural modifications will need a mechanism to explain their recommendation (as per XAI) or generate trust (see Pillar 5) (61). Trust will possibly be more important where behavior modification is indirect through non-interruptive techniques (e.g. re-ordering preference lists or otherwise adapting the user interface to make the recommended choice more accessible).

## 5. Continuous clinical evaluation

Our analogy with translational medicine breaks down at the evaluation stage. For drug discovery, evaluation is *via* a randomized controlled trial (RCT). Randomization handles unanticipated bias and ML4H should hold itself to the same standard but of 350,000 studies registered on ClinicalTrials.gov in 2020, just 358 evaluated ML4H, and only 66 were randomized (62). As usual for ML4H, those RCTs were not interacting with EHR data. They were evaluations of algorithms supporting imaging, cataract screening, colonoscopy, cardiotocographs and more (63–69).

Where the ML4H intervention delivers a novel biological treatment strategy, then it is appropriate to reach for the full paraphenalia used in Clinical Trials of Investigational Medicinal Products (CTIMPs) (2). But in many cases, algorithms will be used to optimize operational workflows and clinical pathways. These pathways may be specific and contextual rather than generalizable. Poor external validity is not a critique: an algorithm that is useful or important in one institution does not have to be relevant in the next (the “myth of generalizability”) (70). Moreover, the algorithm is not the same as the patented and fixed active ingredient in a medicinal product. This is no single point in time nor single host environment at which it can be declared enduringly effective. This means that institutions deploying and relying on these tools need a strategy for rapid continuous clinical and operational evaluation.

This time the EHR may provide an advantage instead of just additional complexity. Since ML4H algorithms must be implemented through some form of direct or indirect CDSS, then the next logical step is to randomize the deployment of those alerts. This in itself is not novel. Randomized deterministic alerts from CDSS are part of the standard evaluation toolkit for quality improvement initiatives in at NYU Langone (71), and for research elsewhere (72). At NYU Langone, such tooling permitted a small team to deliver 10 randomized trials within a single year (71).

The final pillar in our CDE uses the same approach for the probabilistic insights derived from ML4H. Excellent patient and public involvement, and ethical guidance, will be required to distinguish those algorithms that require per patient point-of-care consent from those that can use opt-out or cluster methods. But we think that latter group is large for two reasons. Firstly, patients are exposed to varying treatment regimes by dint of their random interaction with different clinicians based on geography (the healthcare provider they access) and time (staff holidays and shift patterns etc.). This routine variation in practice is summarized as the 60-30-10 problem: 60% of care follows best practice; 30% is wasteful or ineffective and 10% is harmful (6). Secondly, because the intervention is informational, there is ethical precedent for patient level randomization without consent (e.g. Acute Kidney Injury alerts) (72). This hints at a larger and more routine role for randomization in evaluation of algorithms. This in turn is supported by a growing (52, 73, 74) but sometimes conflicting (75) literature on opt-out consent in Learning Healthcare Systems (LHS). As such, progress will require careful attention to a range of concerns.

At our own institution, we have extended this ethical and safety case one step further, and we are piloting a study design where the randomization is non-mandatory: a nudge not an order (76). The clinician is explicitly invited to only comply with the randomization where they have equipoise themselves. Where they have a preference, they overrule the alert (see Vignette 1 in the **Supplementary Material**).

Embedded randomized digital evaluation should permit rapid evidence generation, and build the trust needed to support the implementation described under Pillar 4.

## Drug discovery parallels

We have described a template for a Clinical Deployment Environment that supports the translation of ML4H algorithms from bench to bedside. Although the requirements differ, the objective is similar to that for drug development. A similar approach to phasing has previously been proposed for (biomarker) prediction models (77).

Most ML4H that derives value from the EHR is in the pre-clinical phase. In drug development, the objective of this phase is to identify candidate molecules which might make effective drugs. Evaluation is conducted *in vitro*. Metrics used to evaluate candidates, such binding affinity or other pharmacokinetic properties, describe the properties of the molecule (78). For ML, the objective is to identify candidate algorithms, comprising of input variables and model structures, which might make the core of an effective CDSS. Evaluation is conducted offline on de-identified datasets. Metrics used to evaluate candidates, such Area Under the Receiver Operator Curve (AUROC), the F1 score and calibration, describe the properties of the algorithm (79).

Phase 1 drug trials are the first time a drug candidate is tested in humans. They are conducted in small numbers of healthy volunteers. The aim of the trial is to determine the feasibility of progressing to trials in patients by determining drug safety and appropriate dosage. Drug formulation, the processes by which substances are combined with the active pharmaceutical ingredient to optimize the acceptability and effective delivery of the drug, is also considered at this stage. Phase 1 ML4H trials are the first time an algorithm candidate is tested within the healthcare environment. The aim of the trial is to determine the feasibility of progressing to trials of efficacy by ensuring the algorithm implementation is safe, reliable and able to cope with real-world data quality issues. The development of a mechanism to deliver of algorithm outputs embedded in the clinical workflow is also be considered at this stage.

Phase 2 drug trials involve recruitment of small numbers patients with the disease of interest, typically 50–200. The aim is to determine drug efficacy at treating the disease. Treating clinicians are involved in so far as they must agree to prescribe the drug for their patients. The trials are often too short to determine long term outcomes, therefore surrogate measures such biomarker status or change in tumour size are used as endpoints (80). Phase 2 ML4H trials involve recruitment of small numbers of clinicians making the decision of interest, typically 5–10. The aim is to determine the efficacy of the algorithm in improving their decisions. Patients are involved in so far as they must agree to be on the receiving end of these supported decisions and identifiable data is required. Endpoints are markers of successful task completion in all cases. Investigations to determine ways in which the system could be more successful in influencing user behavior are carried out at this stage. These include usability analyses, considerations of how well the ML4H/CDSS is integrated into the overall system and implementation studies to identify how best to optimize end-user adoption and engagement (57).

Phase 3 drug trials involve the recruitment of large numbers of patients to determine whether a drug is effective in improving patient outcomes. The gold standard of trial design is a double-blinded randomized controlled trial (RCT). Phase 3 ML4H trials will require integration of data from multiple centers for algorithms acting on specific decisions but inevitably adapted to their local data environment.

The phases of drug development are not meant to be matched 1:1 to the pillars of the CDE described here: in fact, our argument for “real-world” *development* deliberately seeks to merge the steps. But the parallel is drawn to highlight the effort necessary to see ML4H have an impact on the clinical and operational decision making in the workplace. Heretofore this effort has been hugely underestimated.

## Conclusion

Even this analogy stops short of the full task of deployment. With drug development, the universities and the pharmaceutical industry go on to take advantage of a supply chain to deliver the drug to the hospital with the necessary quality control and monitoring. Those prescribing and administering the drug have spent years in training, and are supported by pharmacists and medication safety experts. And even after the drug is administered, observation and long term follow-up continue to identify side-effects and long term hazards.

That network of expertise and infrastructure is largely in place where software *is within* (not *as*) a medical device, but is only just being envisioned where the data driving ML4H comes from the EHR. This distinction needs to be made else the disillusionment with the promise of ML4H will continue. The technology does have the potential to change how we deliver health but the methodology alone is insufficient. The impressive demonstrations of the power of AI and ML to beat humans in games, and predict protein structures does not mean that these tools are ready for wide spread deployment.

But we should not be pessimistic. As per author William Gibson, it is clear that “The Future Has Arrived — It”s Just Not Evenly Distributed.” Beyond healthcare, machine learning has already demonstrated that it can reliably create value (5). It is now our responsibility to take those lessons and adapt them for our patients.

The Five Pillars outlined here are a sketch of that redistribution. They are born from our local experience (Pillars 1, 2 and 5) and our wider observations (Pillars 3 and 4). They fundamentally are an argument for a professionalization of ML4H, and a caution against the “get-rich quick” headlines in the popular and scientific press (1). We envision a future where each algorithm is managed in a digital pharmacy with the same rigor that we apply to medicines. But unlike drugs, some of these algorithms will have their entire life-cycle, from development to deployment, managed by the local healthcare provider. Computer vision tasks that support diagnostic radiology can be partially developed offline. Components of sepsis prediction tools will transfer from institution to institution but will need adapting to local clinical workflows. But there will be opportunity and value for ML4H to optimize operational tasks that are temporary or specific to that institution. This means that some development and much of the deployment will require a suitably trained workforce, and an infrastructure perhaps supported by these five pillars.

## Author contributions

All authors listed have made a substantial, direct and intellectual contribution to the work, and approved it for publication. All authors contributed to the article and approved the submitted version.

## Footnotes

^1^One infamous example from the financial services sector saw a firm lose 170, 000*persecond*(*morethan*400m in 45 min) when an outdated piece of code leaked into production. The firm in question was fined a further <$>12m for “inadequate safeguards” allowing “millions of erroneous orders” (25).
