# Supplementary material for: Clinical deployment environments: Five pillars of translational machine learning for health
Source: Front Digit Health. 2022 Aug 19;4:939292. doi: 10.3389/fdgth.2022.939292 (PMC9437594; doi:10.3389/fdgth.2022.939292)
Supplement: Supplementary file 1 [file Data_Sheet_1_v1.pdf]

Supplementary Material

1 EMAP DESIGN PRINCIPLES

Our real-world development is performed on the Experimental Medicine Application Platform (EMAP). EMAP is a clinical laboratory within which ML4H researchers can iteratively build, test and gather feedback from the bedside. It unifies the data and the tools for off-line and online development of ML4H models.

1.1 Live data using HL7v2

We used HL7 (version 2.3+) rather than FHIR as our primary means of access to the EHR because the standard is open, the messages are real time, and the infrastructure already exists even in digitally immature hospitals. A similar approach was used to build a statewide clinical data warehouse in South Carolina spanning multiple institutions and merging data for millions of patients.(Turley, 2016)

Within a hospital, rather than multiple direct pair-wise connections between each system, HL7v2 messages are routed through an integration or interface engine.(Jacob, 2008) Integration engines are a core component in most if not all digital health systems. They orchestrate message routing from source to one or more consumers, they manage the message queues, and because HL7v2 standards are loose they often translate messages to ensure compatibility between systems.

1.2 Sandboxed

This is advantageous because it creates a single point of contact, and reduces installation and maintenance costs. Moreover, because messages were pushed (rather than pulled) from the live clinical system, the work load remains under the control of the source system. Downstream applications are unable to escalate demand to upstream systems. This last point means there is minimal additional burden on clinical systems (the message are already being generated) thus reducing deployment risk.

1.3 Code-to-data

Live data brings an orthogonal but important secondary benefit in that it inverts the data-to-code paradigm.(Guinney and Saez-Rodriguez, 2018) Integrating development and deployment mandates a platform within the same security envelope as the EHR. This code-to-data approach avoids many of the well known challenges of data sharing that troubles health data research.(Powles and Hodson, 2017) Instead, developers come to work with data under the same controls and protections as the original record.

We argue that this approach appropriately balances risks and benefits. In particular, whilst there is greater risk delegating responsibility to individuals, this is mitigated through the benefits of a responsive development environment. Rather than extracting and attempting to anonymise health data, and moving these data to externally located researchers, we bring the researchers to the data. Enabling ’code to data’ rather than relying on ’data to code’ allows us to draw on all of the ’Five Safes’: safe data, safe people, safe settings, safe projects, and safe outputs.(Ritchie, 2017)

Both EMAP databases and the development environment are hosted within the hospital firewall (private cloud). Users are under the same governance process as clinical and other hospital staff. There is no external access. Authorised developers may work with access to PII for application deployment but research teams

work with a de-identified. Deployed applications integrate the PII with the ML4H model and display predictions to clinical teams in context.

1.4 Developer ergonomics

Our third requirement focuses on developer ergonomics. A ’code-to-data’ approach is not feasible if the necessary tooling is not available in the health care environment. This means providing parity in the development environment with regards to tools and workflow.

1.4.1 Application Development Environment

Whilst it is possible to deploy a static set of analytic and development tools with relative ease (i.e. a standalone statistical analysis package), this does not support a modern data science or application development workflow. Such workflows assume free access to install new tools and update dependencies, to collaborate through version control platforms, and to perform continuous integration testing using external infrastructure.

We provide a Linux based virtual machine hosted within the hospital digital environment. The user is not given root access (i.e. full control) but the machine is adapted to support the deployment of applications hosted within docker containers. Network access, filtered by an HTTP proxy, permits the build and orchestration of docker containers. Version control is available locally through a git server, and appropriately certified users are able to use external version control tools. Filters automatically exclude files identified as likely to contain data, and private repositories are preferred by default. Applications are able to verify user identity against the hospital’s active directory so that web applications can be authenticated using existing credentials.[1]

1.4.2 SQL: unified interface for as wide an audience as possible

Most researchers expect to access data as flat tables, or from a relational database. The learning curve for using FHIR is not steep, but it is not designed for bulk queries nor is it able to handle ad-hoc joins or aggregation.(**?**) Flat (bulk) FHIR is not mature, nor designed for live interactions.(202, 2021) This means that training and prediction, and development and deployment would require separate workflows, skills and potentially staffing. We therefore specified a single data source for both training and prediction, and chose SQL as the interface with the widest group of users in health care. This has the added benefit of the platform being accessible to business intelligence and audit teams as well as ML4H developers.

2 EMAP DATA FLOWS

1. An additional interface is connected to the hospital HL7 integration engine that consumes a subset of all messages.
2. These messages are copied to a PostgreSQL database called the Immutable Data Store (IDS). Each raw message is stored with a unique message identifier and a small amount of metadata (e.g. message source, message type, message timestamp). With the IDS, it becomes possible to replay messages from any point in time, and therefore rebuild at will any downstream structures.

[1] A typical set-up would see the team run a Jupyter Lab server within a docker container on that machine with shared file access to permit collaboration between team members. The ML4H researcher would work from this environment using separately managed credential to access live and static data stores. An application developer would orchestrate dockerised applications as needed to support the researcher. These might include web applications to return the model results directly to the clinical team for evaluation, or a services that would provide a suitable API for the results to be called from the EHR. This delegated responsibility strikes a balance between security, and a responsive development environment.

3.The **message-reader** processes the live messages as they appear in the IDS. It converts each message to an in-house interchange format that is coded as a Java package involving a set of serialisable Java classes, serialised test messages for integration and system testing and helper methods for testing. The format has been designed to formalise custom HL7 implementation semantics into use-specific fields, allowing the processing of these messages downstream to be ignorant of HL7.

4.Separately, a **table-reader** performs the same job as the message-reader but working from one or more databases rather than the message store. This permits access to historical data that was shared prior to the g̈o-live… date of any particular feed. It also allows ingestion of data that is not yet being shared through the integration engine. Both the message-reader and the table-reader convert to the same interchange format.

5.Messages, in the interchange format, are batched and sent to an appropriate queue managed by a RabbitMQ server. Priority is given to messages originating with the live stream. Each queue has a maximum number of messages that are allowed, and the services publishing to the queues implement an exponential back off policy to limit the amount of disk space used by the queues.

6.A **event-processor** receives and processes messages from the RabbitMQ server. Processing involves managing a set of identifiers that allows each data item from the message to be linked to a patient (via a medical record number [MRN]) and the healthcare encounter. Specific issues that are managed by the event-processor include:

1.*Inferring context*: HL7 messages are normally sent with a single purpose: to notify an admission, or discharge, request a laboratory test or update a result. However, each message contains segments of contextual information including demographics, and context about that hospital encounter. This means that we can infer that a patient was admitted from a discharge message, or a test was ordered from a result message, and so on. We use this b̈y standerïnformation to construct as complete a view of the current and ongoing state of the hospital as possible.

2.*Messages arriving out of order*: Messages will sometimes arrive out of order. For example, a discharge before an admission. The **event-processor** infers context where necessary to make sense of these situations, and then corrects and updates when prior information is made available at a later time.

3.*Patients having more than one MRN*: Patients may inadvertently re-register with the hospital because sufficient information is not available to match to an existing record or because erroneous information blocks a match. The records will eventually be flagged for a merge, and the **event-processor** will both update the stored identifiers and maintain an audit record of the change.

3 ML-0PS DESIGN PRINCIPLES

Our ML-Ops platform is named FlowEHR: a working prototype supports the deployment and maintenance of a handful of local operational models.(**?**) FlowEHR supports the ingestion of live data from EMAP (above) alongside retrospective data from reporting data warehouses. FHIR will be supported once generally available. We augment the platform with external data sources (e.g. to provide population level priors or regional or national COVID data). The raw input data is automatically monitored and compared against expected distributions to defend against data drift and ensure first line data quality.

FlowEHR also implements an emerging ML architectural pattern known as a *feature store*. Although popularised by hyper-scale technology giants, the feature store is not about data volume but rather offers a solution to data consistency and data reuse problems, both of which act as major barriers to collaboration and iterative model improvement. The feature store provides three important capabilities:

1.**Feature generation**: our researchers and developers collaborate in a deliberate 'pairing' to implement code to transform data into usable features in a consistent and testable manner for both training and

prediction. We argue that it is crucial for data used in training and prediction to have undergone the identical processing steps to avoid difficult to diagnose performance issues (e.g. train/serve skew).(Breck et al., 2019) Generated features are also monitored which provides a second line data quality defence.

2. **Feature storage**: the platform persists the current state of features as well as a record of historical point-in-time correct versions of all features. This is alongside an auditable repository of metadata such as feature versions, data lineage, and usage tracking. These attributes ensure reproducibility, facilitate reuse of common features between teams, and improve safety by providing a mechanism for audit.(Falco et al., 2021)
3. **Feature serving**: the platform exposes a consistent API with separate interfaces for serving large batches of historical data for offline training, and vectors of the latest feature values needed for prediction. Providing separate read pathways enables dynamic de-identification of offline training data used by our researchers while ensuring the live data (containing PII) has undergone the exact same feature transformations.

FlowEHR extends the emphasis on developer ergonomics by providing an access-controlled notebook-style data science environment based on industry standard open-source tooling for the ML modelling lifecycle. The environment provides a simple and easy way to log experiment runs and artefacts alongside hyper parameter strategies and model versions. A structured workflow provides users with sufficient guidance to ensure reproducibility and avoid 'spaghetti code' common in research teams but dangerous for deployment.(Pizka, 2004)

In addition to the feature store, FlowEHR provides an auditable model store for those models that graduate to production. These models are deployed in a decoupled manner using modern container technology and web APIs to allow outputs to be consumed by different services. Once again continuous monitoring guards against model drift and guides retraining decisions.(Davis et al., 2019)

4 NUDGE RANDOMISATION VIGNETTE: ELECTROYTE OPTIMISATION STRATEGY TO PREVENT NEW ONSET ATRIAL FIBRILLATION

Consider the following worked example. We train an algorithm to optimise electrolyte supplementation in critically ill patients at risk of new onset atrial fibrillation (NOAF). We have already observed significant variation in the propensity of the clinical team to administer supplemental magnesium and potassium. Our algorithm evaluates the risk of NOAF in real-time based on existing electrolyte levels, medications, disease type, and the patient's co-morbidities. We have evidence that such supplementation is impactful for cardiac surgery patients(Gu et al., 2012) but no evidence that either our algorithm works nor that the existing evidence generalises to other patient populations. Our model is now used to drive a CDSS that operates in two layers. Firstly, where electrolytes are outside existing (evidence based guidelines) the CDSS makes a strong deterministic recommendation. Secondly, where electrolytes are within the window of the broader guideline but could be optimised, the CDSS makes a nudged randomised recommendation based on the model's prediction.

During the pilot phase the study might run with pre-emptive consent, but if the pilot demonstrates safety and acceptability (for the methodology rather than the intervention) then it justifiable to transition to opt-out consent. This allows the trial to scale, and treatment effects to be estimated as per any RCT with imperfect compliance.(Wilson et al., 2022)

Here the nudge is directly linked to the treatment allocation via the CDSS, but randomised indirect nudges without CDSS are also possible.(Chen et al., 2022) These might alter how information and choice

is presented within the EHR by altering the ordering of investigations in a pick list. Such approaches have already demonstrated efficacy for diagnostic, screening and monitoring test orders.(Main et al., 2010; **?**)

REFERENCES

Turley CB. Leveraging a statewide clinical data warehouse to expand boundaries of the learning health system. *eGEMs (Generating Evidence & Methods to improve patient outcomes)* **4** (2016) 25.

Jacob B. Mirth: Standards-based open source healthcare interface engine (2008).

Guinney J, Saez-Rodriguez J. Alternative models for sharing confidential biomedical data. *Nature biotechnology* **36** (2018) 391–392.

Powles J, Hodson H. Google DeepMind and healthcare in an age of algorithms. *Health and Technology* **7** (2017) 351–367. doi:10.1007/s12553-017-0179-1.

Ritchie F. The 'Five Safes': a framework for planning, designing and evaluating data access solutions (2017).

FHIR bulk data access (flat FHIR) (2021).

Breck E, Polyzotis N, Roy S, Whang S, Zinkevich M. Data validation for machine learning. *Data validation for machine learning.* (2019).

Falco G, Shneiderman B, Badger J, Carrier R, Dahbura A, Danks D, et al. Governing AI safety through independent audits. *Nature Machine Intelligence* **3** (2021) 566–571.

Pizka M. Straightening spaghetti-code with refactoring. *Straightening spaghetti-code with refactoring*, vol. Software Engineering Research and Practice (2004), 846–852.

Davis S, Greevy R, Fonnesbeck C, Lasko T, Walsh C, Matheny M. A nonparametric updating method to correct clinical prediction model drift. *Journal of the American Medical Informatics Association : JAMIA* **26** (2019) 1448–1457.

Gu W, Wu Z, Wang P, Aung L, Yin R. Intravenous magnesium prevents atrial fibrillation after coronary artery bypass grafting: a meta-analysis of 7 double-blind, placebo-controlled, randomized clinical trials. *Trials* **13** (2012) 41.

Wilson MG, Asselbergs FW, Harris SK. Learning from individualised variation for evidence generation within a learning health system. *British Journal of Anaesthesia* (2022).

Chen Y, Harris S, Rogers Y, Ahmad T, Asselbergs F. Nudging within learning health systems: next generation decision support to improve cardiovascular care. *European heart journal* **43** (2022) 1296–1306.

Main C, Moxham T, Wyatt JC, Kay J, Anderson R, Stein K. Computerised decision support systems in order communication for diagnostic, screening or monitoring test ordering: systematic reviews of the effects and cost-effectiveness of systems. *Health Technology Assessment* **14** (2010). doi:10.3310/hta14480.
